# Genome Shows no Recent Inbreeding in Near-Extinction Woolly Rhinoceros Sample Found in Ancient Wolf's Stomach

**DOI:** 10.1093/gbe/evaf239

**Published:** 2026-01-14

**Authors:** Sólveig M Guðjónsdóttir, Edana Lord, Zoé Pochon, Špela Lemež, Nicolas Dussex, David W G Stanton, Mikkel-Holger S Sinding, Sergey Fedorov, Love Dalén, J Camilo Chacón-Duque

**Affiliations:** Centre for Palaeogenetics, Stockholm 10691, Sweden; Centre for Palaeogenetics, Stockholm 10691, Sweden; Department of Zoology, Stockholm University 10691, Stockholm, Sweden; Centre for Palaeogenetics, Stockholm 10691, Sweden; Department of Archaeology and Classical Studies, Stockholm University, Stockholm 11418, Sweden; Centre for Palaeogenetics, Stockholm 10691, Sweden; Centre for Palaeogenetics, Stockholm 10691, Sweden; Department of Population Analysis and Monitoring, Swedish Museum of Natural History, Stockholm 10405, Sweden; Centre for Palaeogenetics, Stockholm 10691, Sweden; School of Biosciences, Cardiff University, Cardiff CF10 3AX, UK; Department of Birds and Mammals, Greenland Institute of Natural Resources, 3900 Nuuk, Greenland; Mammoth Museum of North-Eastern Federal University, 677000 Yakutsk, Russia; Centre for Palaeogenetics, Stockholm 10691, Sweden; Department of Zoology, Stockholm University 10691, Stockholm, Sweden; Centre for Palaeogenetics, Stockholm 10691, Sweden; Department of Zoology, Stockholm University 10691, Stockholm, Sweden

**Keywords:** woolly rhinoceros, extinction, ancient DNA, genomic erosion

## Abstract

Using temporarily spaced high-coverage ancient genomes, we can assess population decline prior to extinction. However, finding suitable ancient remains for recovering this type of data is challenging. Here, we sequenced a high-coverage genome from muscle tissue of a 14,400-year-old woolly rhinoceros (*Coelodonta antiquitatis*)—a cold-adapted herbivore that went extinct ∼14,000-years ago—found inside a permafrost-preserved wolf's stomach. We compared genome-wide diversity, inbreeding, genetic load, and population size changes in this sample with two other Late Pleistocene Siberian woolly rhinoceros. We found no evidence of population size decline, nor any genomic erosion, shortly prior to the species' demise. Given the few long homozygous segments, typically indicative of recent inbreeding, we infer a stable population size only a few centuries before extinction. Thus, the woolly rhinoceros' extinction likely happened rapidly, during the Bølling–Allerød interstadial. This study demonstrates the ability to recover high-quality DNA from unlikely sources to elucidate species' extinction dynamics.

Significance The woolly rhinoceros went extinct around 14,000 years ago, but little is known about their population decline prior to extinction. We generated a high-coverage genome from one of the last known woolly rhinoceros remains, which was recovered from the stomach contents of a mummified wolf puppy found in the permafrost in Siberia. Combined with two other Late Pleistocene woolly rhinoceros genomes, our results suggest that the population size was stable and there is no genomic signature of recent, rapid population decline close to the species extinction, in contrast to other extinct species and currently endangered species undergoing population decline. Given the scarcity of animal remains close to their extinction times and other key evolutionary events, this study provides a new avenue to obtain high-quality genomic information from unlikely sources.

## Introduction

In the current biodiversity crisis driven by anthropogenic climate change, it becomes increasingly important to understand the underlying drivers of population declines and the propensity of species to go extinct. Prior to extinction, species generally display a reduction in both population size and geographic range, leaving them more vulnerable to stochastic environmental, demographic, and/or genetic events (Melbourne and Hastings 2008). The main concern at the genetic level is that small populations are more vulnerable to genetic drift and increased inbreeding (Frankham 2005). These factors can lead to the loss of genomic diversity and the increase of both homozygous segments and genetic load—referred to as genomic erosion (Bertorelle et al. 2022), which increases the chances of further population decline due to reduced fitness (Charlesworth and Charlesworth 1999). With temporally spaced genomic data, it is possible to track changes in those genomic parameters and gain insight into species population history, especially during decline toward extinction (Diez-del-Molino et al. 2018; von Seth et al. 2021; Jensen and Leigh 2022). The mode of such decline depends on life history traits, demographic dynamics, and the magnitude of stochastic events (Purvis et al. 2000). While some species show a steady decline in population size and persist at low levels for a long time (Dussex et al. 2021; Morin et al. 2021; Pečnerová et al. 2024), others seem to decline rapidly (Zhou et al. 2013; Robinson et al. 2019; van der Valk et al. 2019). It is important to connect the species' evolutionary history and rate of decline to inform the genomic consequences of decline on a population or species.

To generate temporal genomic datasets, it is essential to recover high-quality DNA from ancient organisms. Although it has become easier to recover ancient genomic data (Palkopoulou et al. 2015; Bruniche-Olsen et al. 2018; Lord et al. 2020; Sharko et al. 2021), DNA recovered from these samples is generally of low quality and quantity—a consequence of post-mortem DNA damage and contamination from environmental sources. This has hindered the retrieval of high-coverage (>10× depth of coverage) genomes from ancient samples, which are essential to analyze genome erosion, since it is necessary to accurately infer genotypes to assess genomic variation at the individual level (Günther and Nettelblad 2019; Kutschera et al. 2022). However, recent studies have published high-coverage genomes from a range of Late Pleistocene samples, predominantly, but not limited to, permafrost-preserved megafauna (Barnett et al. 2020; Lord et al. 2020; Wang et al. 2022; Dehasque et al. 2024; Pečnerová et al. 2024).

One iconic megafauna species is the woolly rhinoceros (*Coelodonta antiquitatis*), a cold-adapted herbivore widespread across northern Eurasia until its extinction ∼14,000 years ago (ka) (Stuart and Lister 2012). Its range contracted gradually toward the east from ∼35 ka, likely due to unfavorable environmental conditions in western Europe (Allen et al. 2010; Stuart and Lister 2012). The woolly rhinoceros persisted in northeastern Siberia and displayed complex shifts in its range in response to environmental changes until it disappeared from the fossil record. Additionally, previous paleogenomic analyses of the woolly rhinoceros did not find any indication of recent inbreeding in individuals dated to 18.4 ka (Lord et al. 2020) and 48.5 ka (Liu et al. 2021). Thus, it was concluded that the woolly rhinoceros decline toward extinction occurred rapidly sometime after 18.4 ka, likely associated with the climatic conditions of the Bølling–Allerød interstadial (14.7 to 12.8 ka). However, no whole genome data to date has been recovered from woolly rhinoceros closer to their extinction.

While Late Pleistocene remains of woolly rhinoceros are numerous, very few remains exist from around the estimated time of extinction. However, the mummified remains of a juvenile wolf (*Canis lupus)* were found in the Tumat region of northeastern Siberia (Kandyba et al. 2015; Bergström et al. 2022), and upon dissection a piece of intact mummified tissue was discovered in its stomach (Fig. S1). After DNA extraction and sequencing, the tissue was revealed to be from a woolly rhinoceros and was radiocarbon dated to 14.4 ka, making it one of the youngest known woolly rhinoceros remains (here on referred to as Tumat_14k). Lord et al. (2020) recovered a full mitogenome from the sample, indicating its potential for future whole-genome studies. Here, we obtained a high-coverage genome from this tissue sample, offering an unprecedented opportunity to investigate the extinction process of the woolly rhinoceros. We not only demonstrate the feasibility of recovering high-coverage genomes from low-quality samples but also the usefulness of individual genomes to gain insights into the evolutionary history of an extinct species.

## Materials and Methods

### Sample Information

The mummified tissue (Tumat_14k) was found in the stomach of a preserved wolf puppy recovered from the permafrost in Tumat, a locality of Yakutia in northeastern Siberia, Russia (Kandyba et al. 2015; Bergström et al. 2022). Both the mummified tissue and the wolf puppy have been radiocarbon dated to 14.4 ka (Lord et al. 2020; Bergström et al. 2022) (calibrated using the IntCal20 calibration curve (Reimer et al. 2020) in OxCal v.4.4 (Ramsey and Lee 2013)).

**Fig. 1.**
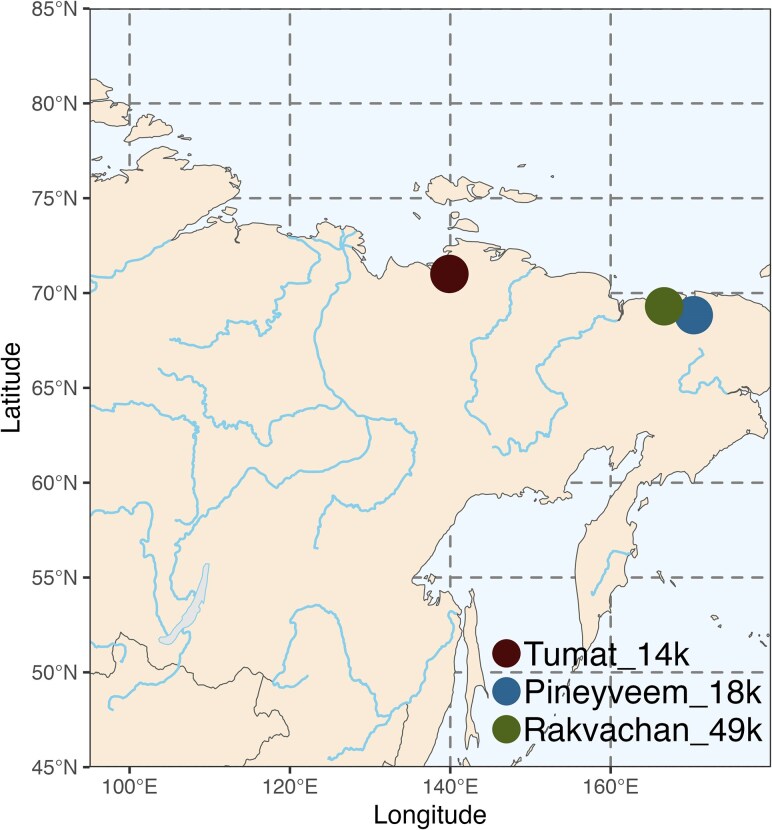
Sample locations in northeast Siberia.

In addition, we computationally reanalyzed two previously published high-coverage woolly rhinoceros genomes Pinevyeem_18k (Lab ID: ND035; ENA: SAMEA6246871) (Lord et al. 2020) and Rakvachan_49k (Lab ID: ND036; NCBI SRA: SAMN17167289) (Liu et al. 2021). Raw data for ND036 were retrieved from NCBI GenBank using SRA toolkit v3.0.3 (http://www-ncbi-nlm-nih-gov.ezp.sub.su.se/books/NBK158900/) with the prefetch function. These samples are from North Chukotka, Russia (Fig. 1). The samples had previously been radiocarbon dated and calibrated using IntCal13 (Lord et al. 2020), but we recalibrated the C14 dates using IntCal20 (Reimer et al. 2020) calibration curve in OxCal v.4.4 (Ramsey and Lee 2013). The calibrated median, including 95% CI, was used (Table S2).

### Sample Processing of Tumat 14k

Before processing the tissue for DNA extraction, it was around 4 cm × 3 cm (Fig. S1). First, a fragment was cut from one corner (Extract B), as was done for the original DNA extract (Extract A) sequenced by Lord et al. (2020). However, Extract B yielded low levels of DNA compared to Extract A (see next section). For this reason, the tissue was divided in half to expose the interior, and 20 small pieces of similar size and weighing ∼15 mg were cut (for DNA Extracts C-V).

### DNA Extraction and Genomic Libraries Preparation

We extracted DNA from the 21 tissue fragments described above following Dabney et al. (2013). Since the first DNA extract (Extract B; Table S1) yielded low endogenous DNA and complexity levels, for the remaining 20 extracts, we optimized the protocol for tissue samples, using the digestion buffer and incubation times described in Gilbert et al. (2007). Double-stranded Illumina libraries were prepared following Meyer and Kircher (2010) as modified by Dehasque et al. (2022), using Min-Elute spin columns (Qiagen) for the clean-up steps and reaction volumes halved. We performed USER treatment where uracils caused by cytosine deamination are removed to account for post-mortem DNA damage (Briggs et al. 2010). From these stock libraries, double-indexed libraries were prepared prior to sequencing. For Extract B, we indexed 6 libraries, with 14 polymerase chain reaction (PCR) cycles during indexing. Given that the results yielded a low complexity, for the remaining 20 extracts, we determined the optimal number of PCR cycles needed to amplify each library during the indexing step without generating an excess of PCR duplicates with the Cq values obtained from a quantitative PCR (qPCR) (Meyer et al. 2008).

A visual inspection of each indexed library for the 20 extracts (Extracts C-V), run on a 1% electrophoresis gel, revealed similar band strength, so the libraries were pooled together, assuming an equimolar concentration. Purification and size selection of the DNA pool were done using magnetic Agencourt AMPure XP beads (Beckman Coulter). For the removal of by-products from the PCR step, such as primer dimers, we added a 1.8× beads-to-pool ratio, placed it on a magnetic rack, and discarded the supernatant. This step was repeated twice to ensure thorough removal. To remove long fragments, likely to be contaminating DNA, we used a 0.5× beads-to-pool ratio and retained only the supernatant.

### Sequencing

#### Quantification and Sequencing

The concentration of the cleaned pool was assessed using a high-sensitivity chip on the Bioanalyzer 2100 (Agilent) and then sent for an initial sequencing, aiming for 1.9 billion reads on one lane of Illumina NovaSeq sequencing platform (paired-end, 2 × 150 bp) at the National Genomics Infrastructure (NGI), Stockholm. This first round of sequencing was used to assess the extracts' DNA quality and endogenous DNA content.

Based on the results from sequencing round one, we selected ten extracts (Text S1) and prepared them for the second round of sequencing following the same laboratory methods described above, with a few modifications. We first generated an additional library from the extracts to increase DNA complexity, resulting in two libraries per extract. Next, we generated 9 to 10 PCR amplifications for each library, completely exhausting their contents and visually inspected the concentration with gel electrophoresis. This resulted in 190 amplified and indexed libraries, which were subsequently pooled together in library pools of 90 to 100 µl. Short and long fragments were cleaned from each pool twice and, after assessing their concentration, were all pooled together based on the number of reads desired from each extraction (Text S1). Based on the data from the first sequencing round, the average DNA fragment size of aligned sequences was below 100 bp, and for this reason, the final pool was sent for sequencing on a full S4 flow cell of Illumina NovaSeq sequencing platform with a paired-end 2 × 100 bp sequencing strategy at NGI, Stockholm.

### Data Processing

#### Alignment of Sequencing Data

Raw sequencing data for Tumat_14k, Pineyveem_18k, and Rakvachan_49k were aligned and processed following the GenErode pipeline (Kutschera et al. 2022) written in Snakemake version 7.20.0. GenErode is designed for estimating and comparing patterns of genomic erosion between samples from separate time periods. We followed it as designed for ancient/historical data.

In short, raw FASTQ sequencing files were adapter and quality trimmed using fastp v0.11.0 (Chen et al. 2018) by simultaneously merging overlapped paired-end reads, trimming adapters, and bases with a quality score < 15. Only merged reads ≥ 30 bp were retained to reduce misalignments (van der Valk et al. 2021) and aligned to the reference genome assembly of the Sumatran rhinoceros (*Dicerorhinus sumatrensis;* GenBank: GCA_014189135.1), the woolly rhinoceros' closest extant relative (Liu et al. 2021) using BWA v0.7.17 (Li and Durbin 2009) and processed using SAMtools v1.9 (Li et al. 2009). BWA (aln option) was used for alignment with recommended ancient DNA parameters (-l 16500 -n 0.01 -o 2) (Palkopoulou et al. 2015). Duplicates were then removed from sorted BAM files using SAMtools and a custom Python script (Kutschera et al. 2022), by using both read start and end alignment coordinates. BAM files were then merged per sample (per extract in the case of Tumat_14k) and realigned around indels using GATK IndelRealigner v3.7 (McKenna et al. 2010).

The following data processing and further downstream analyses were performed outside GenErode but following the methods described there (except for the demographic reconstruction analysis, which is not part of GenErode). After checking for nonendogenous contamination per extract in Tumat_14k, extract U was removed due to high levels of wolf DNA (Text S2). Unaligned reads and reads with alignment quality <30 were filtered out for all three samples using SAMtools view (-F 4 -q 30). To reduce error in downstream analyses due to possible duplicates across indexing PCRs, we performed another round of duplicate removal for all three samples. The coverage for all samples was estimated using SAMtools depth with repeat regions in the reference genome masked. Samples Pineyveem_18k and Rakvachan_49k were subsampled down to a coverage of 10.1× to match Tumat_14k, prior to downstream analyses, to avoid any coverage-related biases in downstream analyses with SAMtools view function -s. To further assess potential contamination, a metagenomic screening was performed on the three samples (Texts S2 and S3).

### Downstream Analyses

#### Variant Calling

Variant calling and filtering steps were performed per sample using BCFtools v1.9 (Danecek et al. 2021) and BEDtools v2.29.2 (Quinlan and Hall 2010). In short, variants were called for all three samples independently using BCFtools mpileup and call commands, filtering out reads with alignment and base quality <30 as well as implementing the option -B to reduce false single-nucleotide polymorphism (SNPs) caused by misalignment. The resulting per-sample variant call format (VCF) files were then sorted prior to further filtering steps. CpG sites and repetitive regions were identified in the reference genome following GenErode (Kutschera et al. 2022). These sites were subsequently removed from the VCFs using BEDtools intersect, as methylation on CpG sites often elude USER treatment (Briggs et al. 2010) and the short length of ancient DNA reads make alignments to repetitive regions difficult (Treangen and Salzberg 2012). Indels (insertions and deletions) and SNPs within 5 bp of an indel were removed using BCFtools filter command, as well as sites with genotype quality <30. We also filtered out sites with depth lower than 1/3× and higher than 10× the estimated average coverage, as sites with a coverage too high or too low can create false heterozygous sites. Heterozygous sites with less than 20% or more than 80% of reads supporting a given allele were excluded using BCFtools view with the function -e. Finally, scaffolds corresponding to the X chromosome (Sc9M7eS_1319;HRSCAF=1962 and Sc9M7eS_931;HRSCAF=1475) and the mitogenome (Sc9M7eS_584;HRSCAF=1017, Sc9M7eS_70;HRSCAF=235, Sc9M7eS_938;HRSCAF=1483) were removed with BEDtools intersect.

All downstream analyses were performed in two datasets, one containing transversions only, which is presented throughout the main text, and the other containing all variants, which is described in the supplementary material (Text S4). Transitions were removed to account for cytosine deamination since Rakvachan_49k was not USER-treated.

#### Demographic Reconstruction and Population Structure

We estimated past changes in effective population size (*N*_e_) for samples Tumat_14k and Pineyveem_18k using the pairwise sequentially Markovian coalescent model (PSMC) v.0.6.5 (Li and Durbin 2011), on a per-sample basis. We performed bootstrapping by splitting the chromosome sequences into shorter segments and ran PSMC with the same parameters 99 times and sampling with replacement. The results were plotted in R v.4.2.3 (R Core Team 2021) with the package Hmisc (Harrel 2025). The time was scaled for each sample by calculating *d* = 2µ*Δ*T* using the rate of substitutions (*µ*) as 2.34 × 10^−8^ substitutions/site/generation (Mays et al. 2018) and *T* as the age of the sample in generations, calculated using a generation time of 12 years (Roth et al. 2013) based on estimations for the Sumatran rhinoceros. For the transversion-only dataset, we used a rate of 0.78 × 10^−8^ substitutions/site/generation. To examine population structure, we performed principal components analysis with PLINK (v1.90b7.4) (Purcell et al. 2007). We first converted the merged VCF to plink format and then ran the –pca option.

#### Heterozygosity

Genome-wide heterozygosity was estimated directly from each individual VCF file using BCFtools (-stats option), as the fraction of heterozygous sites compared the total number of sites. Additionally, for the dataset containing all variants, we applied a maximum likelihood approach (mlRho v2.9 (Haubold et al. 2010)) to estimate the population mutation rate *θ* as an approximation to heterozygosity under the infinite sites model.

#### Inbreeding

The individual inbreeding coefficient (*F*_ROH_) was estimated as the proportion of the autosomal genome contained within ROH segments of different lengths (>0.1 Mb (megabases), >0.5 Mb, >1 Mb, and >2 Mb). The different length thresholds were used to differentiate recent inbreeding from background relatedness due to the samples' population histories, since ROHs tend to become shorter with time due to recombination (Curik et al. 2014). We identified ROHs using the sliding-window approach of plink v1.90b6.12 (Purcell et al. 2007). Individual VCF files were merged and both nonbiallelic sites and sites not present in all samples were filtered out with BCFtools. We used the same parameters described by Lord et al. (2020): The sliding window was set to contain 100 SNPs (*−homozyg-window-snp 100)* and defined as homozygous if there were not more than 5 heterozygous sites (*−homozyg-window-het 5*) or 15 missing sites (*−homozyg-window-missing 15*) per window. Furthermore, we identified a SNP as part of a homozygous segment if a minimum of 5% of windows containing it were defined as homozygous (*−homozyg-window-threshold 0.05*). A homozygous segment had to cover a minimum of 100 kb (kilobases) (*−homozyg-kb 100*) to be defined as ROH, containing with no more than 750 heterozygous sites (*−homozyg-het 750*) and at least 25 SNPs (*−homozyg-snp 25*). We tested for differences in the average size of ROHs among the samples for four different size thresholds >0.1 Mb, >0.5 Mb, >1 Mb, and >2 Mb (Text S4).

To replicate the results, we also estimated ROH using BCFtools/ROH (Narasimhan et al. 2016) with default parameters, using genotype calls (-G30) and setting allele frequencies as unknown (−AF-dflt 0.4).

#### Genetic Load

Variants were characterized into different impact classes using SnpEff v4.3 (Cingolani et al. 2012), based on a custom annotation for the Sumatran Rhinoceros from Lord et al. (2020). Variants were defined as high impact (frameshift or loss of function mutations that highly disrupt protein function), moderate impact (nonsynonymous mutations that may disrupt protein function), or low impact (synonymous mutations that are unlikely to disrupt protein function), according to SnpEff. Using SnpSift, the following fields were extracted: CHROM, POS, REF, ALT, GEN [*].GT, ANN [*].IMPACT, and ANN[*].EFFECT from the annotated VCF. Genetic load for each impact class was calculated as: (count of derived alleles of class x, counting homozygous variants as two)/(count of total derived alleles, counting homozygous alleles as two) (Table S6).

#### Derived Variants

To determine whether the nonsynonymous derived alleles identified in Lord et al. (2020) were also present in the additional two woolly rhinoceros genomes, we used a custom Python script to determine the alleles in the BCFs (with and without transitions) at the derived positions previously identified (Table S3).

## Results

### Generating a High-Coverage Woolly Rhinoceros Genome from the Stomach Content of an Ancient Wolf

Lord et al. (2020) estimated the endogenous DNA content of Tumat_14k as ∼10% based on a single extract. We initially generated an additional DNA extract for which we intended to obtain deep sequencing data, but both endogenous DNA content (ie the percentage of sequenced reads mapped to the target reference genome) and DNA complexity (ie the amount of unique DNA molecules in a sequenced genomic library) were low (Extract B, Table S1). To increase the probability of recovering further extracts with good endogenous DNA content and to maximize the complexity, we generated 20 additional extracts from different fragments of the tissue sample. We shotgun sequenced an average of 88 million reads per extract (range: 55 to 150 million) and mapped all sequencing data to the Sumatran rhinoceros (*Dicerorhinus sumatrensis*) reference genome (Lord et al. 2020), the woolly rhinoceros' closest extant relative, with a divergence time of ∼9.3 million years (Orlando et al. 2003; Liu et al. 2021). Endogenous DNA content and complexity were variable across the extracts (range: 1.9% to 8.3% endogenous and 57.6% to 85.1% complexity). We then selected the ten best extracts for further sequencing to maximize the following parameters: (*i*) endogenous DNA content, (*ii*) complexity, and (*iii*) mapping quality scores (Text S1). We also mapped published whole-genome sequencing data from two northeastern Siberian woolly rhinoceros (Pineyveem_18k (ND035); 18.4 ka cal & Rakvachan_49k (ND036); 48.5 ka cal) (Fig. 1). The three samples had a depth of coverage ranging from 10.1 to 11.1× (Table S2). Finally, we note that all three samples share nonsynonymous derived sites that are likely important in the evolution of woolly rhinoceros (Table S3).

### Identification of Nonendogenous DNA Content

Since Tumat_14k was found inside the stomach of a wolf, we estimated the proportion of wolf DNA in all 22 extracts. We mapped the sequencing data from each extract to a concatenated reference containing gray wolf and woolly rhinoceros mitogenomes, as well as other vertebrates that could be potential contaminants, including humans (Text S2). With the exception of one extract, which was removed from all downstream analyses (DS253U), we found that the percentage of wolf mitogenome reads across samples was minimal (average 2.3%; Fig. S2 and Text S2). We also estimated the potential wolf contamination at the nuclear genome level and demonstrated that stringent filtering at the variant calling stage was sufficient to mitigate any contamination (Text S2). Additionally, we performed a metagenomic screening to assess the presence of ancient host-associated microbes and pathogens (Text S3 and Table S4). However, we were not able to identify ancient microbial organisms based on post-mortem DNA damage patterns (limited to CpG sites on the USER-treated samples) and most microorganisms found could be interpreted as environmental contamination. Nevertheless, in Tumat_14k we detected *Carnobacteria* and *Lactobacilli* species, which are generally associated with meat kept in cold environments, and *Clostridia spp.*, *Listeria monocytogenes,* and *Paraclostridium bifermentans,* which are associated with the intestinal tract of animals but are also commonly found in soils (Table S4).

### Demographic History of Late Pleistocene Woolly Rhinoceros

We reconstructed the demographic history of Tumat_14k, contextualizing it with the other two samples and assessing whether its genome could reveal some signatures of population decline associated with the species extinction. After variant calling and quality control (see Methods), we identified approximately 22 million single-nucleotide polymorphisms (SNPs) in the three samples. Since Rakvachan_49k was not enzymatically treated to remove signatures of post-mortem DNA damage (Liu et al. 2021), the reported results are based on a subset of ∼7.4 million SNPs that were retained after filtering out transitions. For Tumat_14k and Pineyveem_18k, we also performed the analysis on the full SNP set, obtaining comparable results (Text S4).

**Fig. 2.**
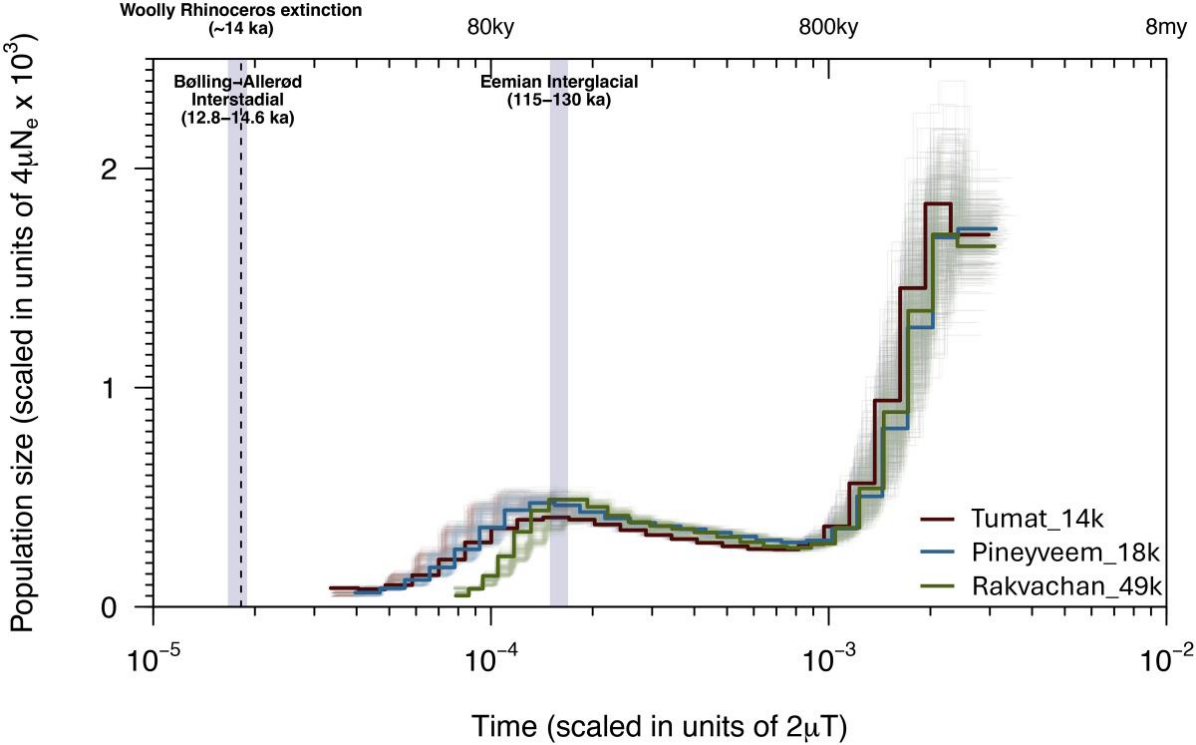
Temporal effective population size (*N*_e_) changes for the three woolly rhinoceros. Lighter shaded lines in the corresponding color show the bootstrap values for each sample. The lower *x*-axis shows time in units of divergence per base pair, while the upper *x*-axis is time in years before present, assuming a transversion-only substitution rate of 0.78 × 10^−8^ substitutions/site/generation and a 12-year generation time. The final 10,000 years were removed from the PSMC. The y-axis shows scaled population size. The two shaded boxes refer to the Eemian interglacial (130 to 115 ka) and the Bølling–Allerød interstadial (14.7 to 12.8 ka). The dashed line represents the proposed extinction date of woolly rhinoceros at ∼14 ka.

To explore changes in population size through time, we used the Pairwise Sequentially Markovian Coalescent (PSMC) approach (Li and Durbin 2011) (Fig. 2). The three samples showed a similar demographic trajectory, with a steep decline during the Early Pleistocene, followed by a stable population size throughout the Middle Pleistocene and a gradual decline during the Late Pleistocene. Rakvachan_49k declined from 114 ka to 63 ka, with a ten-fold reduction in *N_e_* from ∼15,600 to ∼1,600. Tumat_14k and Pineyveem_18k declined slightly later than Rakvachan_49k, from 100 to 109 ka until 34 ka and 33 ka, respectively. We also examined population structure with principal components analysis, with PC1 separating Rakvachan_49k from the other two samples, and PC2 separating the samples by geography (Fig. S3).

### No Recent Inbreeding Close to Extinction

The woolly rhinoceros' genomic history was further analyzed by assessing genome erosion indices prior to extinction. Genome-wide heterozygosity was estimated using allele counts from variant calling and all three samples had ∼0.4 heterozygous sites per 1,000 bp (Table 1). These results were also replicated for Tumat_14k and Pineyveem_18k by estimating the population mutation rate *θ* for all SNPs following a maximum likelihood approach (Text S4).

**Table 1:** Genome erosion parameters for the three woolly rhinoceros

Sample	Genome-wide heterozygosity per 1000 bp	F_ROH >0.1 Mb_	Genetic load per 100k SNPs		
			High impact	Moderate impact	Low impact
Tumat_14k	0.433	7.53%	24	912	738
Pineyveem_18k	0.427	7.45%	24	909	737
Rakvachan_49k	0.430	7.45%	24	910	737

All metrics were conducted on a transversion-only dataset.

**Fig. 3.**
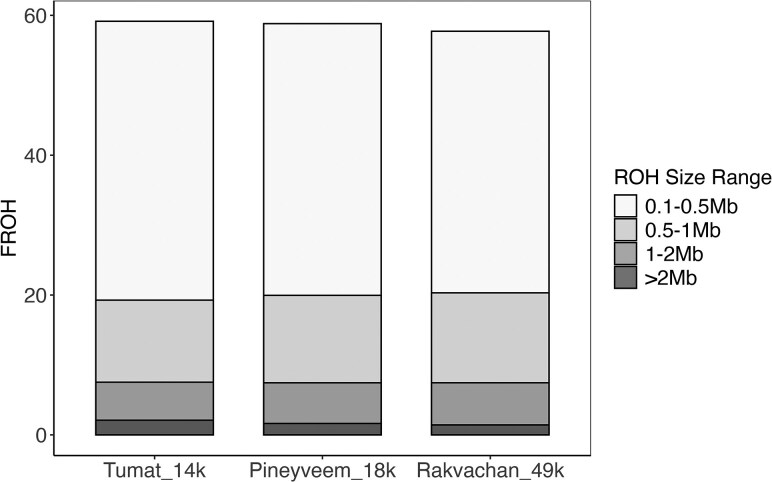
*F*_ROH_ estimates for the three woolly rhinoceros using PLINK after removing transitions.

Additionally, we assessed inbreeding by identifying homozygous segments across the genome, commonly referred to as runs of homozygosity (ROH). The distribution and frequency of ROH >0.1 Mb were similar among all three samples, with the same mean and median length of ROH segments (Table S5). We observed no significant differences in ROH size distribution between the samples comparing four size thresholds: >0.1 Mb, >0.5 Mb, >1 Mb, and >2 Mb (Fig. 3 and Table S5; *P*-value > 0.001). The majority of these stretches of homozygosity were under 1 Mb long (∼98%), with only a few ROH windows (0.3%) over 2 Mb. The longest ROH in Tumat_14k was 8.9 Mb, 4.7 Mb in Pineyveem_18k, and 3.9 Mb in Rakvachan_49k. Considering that the three samples show extremely similar levels of inbreeding despite the notable differences in their ages, we corroborated that the inferred ROH regions are different across the samples, as expected for individuals from populations separated in space and time (Fig. S4).

To further examine the levels of inbreeding, we calculated the inbreeding coefficient (*F*_ROH_), by estimating the proportion of the genome within ROH segments >0.1 Mb. The overall *F*_ROH_ was similar among all samples, with 58% to 59% of their genome within homozygous segments. Comparing *F*_ROH_ values for different size ranges (0.1 to 0.5 Mb, 0.5 to 1 Mb, and 1 to 2 Mb and >2 Mb), we found that each size range was very similar between the samples, including long F_ROH >2 Mb_, which ranged between 1% and 2% (Fig. 3). We replicated these results by inferring ROHs with another widely used statistical approach. Even though this approach inferred higher levels of inbreeding overall, the pattern for the different size ranges remains the same (Fig. S5).

Finally, we examined genetic load using SnpEff (Cingolani et al. 2012) and calculated the number of variants per impact class. We found no difference in genetic load in any impact category (high, moderate, and low) across the three samples (Table 1). To evaluate if the low variation in genetic load between samples could be caused by the exclusion of transitions, we also performed this analysis, including transitions (Text S4). However, the results were similar, indicating no effect of post-mortem DNA damage on this particular set of samples for estimating genetic load (Table S6).

## Discussion

By sequencing a high-coverage genome from a poorly preserved sample of a woolly rhinoceros dated close to the estimated time of the species' extinction based on the fossil record, we were able to get a snapshot of a critical time in the species' history. Recovering high-quality genome data allowed us to confidently call variable sites, and thus, conduct single-sample demographic and evolutionary analyses that provided new insights into the species extinction. Furthermore, we demonstrate the feasibility of recovering high-coverage genomic data from rare and unique samples. By using multiple independent DNA extractions and minimizing the amount of PCR duplicates during the preparation of genomic libraries, it is possible to enhance DNA complexity, thus sequencing efficiently to higher coverage. This serves as an example for other species with a scarcity of samples around key evolutionary events.

The demographic trajectories of all three samples were consistent during the Early and Middle Pleistocene. However, we note that the population history of Rakvachan_49k diverged from the other two samples during the Late Pleistocene. Interestingly, despite having been found in geographical proximity to Pineyveem_18k, this sample belongs to a separate mitogenome clade that diverged around 440 to 116 ka (Lord et al. 2020). Principal components analysis also separates Rakvachan_49k from the other two samples (Fig. S3). This may suggest a partial or total population replacement in the northern Chukotka region during the Late Pleistocene, which should be further investigated with population-level ancient genomes. Our analyses suggest that the population size was stable from ∼30 ka until ∼14 ka. The PSMC indicates no reduction in population size at the onset of the Bølling–Allerød interstadial at ∼14.7 ka (Text S4). While we note that PSMC analyses do not have the power to detect sharp declines close to the end of the curve, the consistency between the heterozygosity and inbreeding estimates of Pineyveem_18k and Tumat_14k does not suggest a drastic population decline took place in this time frame.

Unlike what has been reported for other extinct and endangered species (Palkopoulou et al. 2015; Liu et al. 2021; Sánchez-Barreiro et al. 2021, 2023; von Seth et al. 2021; Pečnerová et al. 2024), we found no evidence of reduced genetic diversity or increased inbreeding and genetic load in Tumat_14k, despite its proximity to the estimated date of extinction. Both genome-wide heterozygosity estimates and the fraction of ROH segments of different sizes are almost identical for all three individuals. Considering that a large proportion of ROH segments are short, distant in time background relatedness is likely the main source of homozygosity, rather than recent mating between closely related individuals. Long ROHs indicative of recent inbreeding would typically span over larger chromosome segments (>2 Mb) (Pemberton et al. 2012), as they have not been broken up by recombination (Curik et al. 2014). The fraction of long ROHs remains consistently low between the woolly rhinoceros genomes, unlike what is observed during the more recent declines of extant rhinos (Sánchez-Barreiro et al. 2021, 2023). Thus, our results support a relatively stable woolly rhinoceros population in Northern Siberia at least until 14.4k. Furthermore, the population decline prior to ∼30k may have purged some of its genetic load, as suggested for other species that experienced long and gradual declines (Morin et al. 2021; Pečnerová et al. 2024).

Given our results, we suggest that any change at the genomic level associated with the species extinction must have taken place during the last few hundred years of the species' existence, or that the extinction was too rapid to leave a detectable genomic erosion pattern. We note that last appearance dates in the fossil record do not exclude the possibility that the species persisted for longer. Sedimentary ancient DNA (sedaDNA) can be a useful tool for examining the disappearance of megafauna, especially in combination with additional environmental proxies (eg Graham et al. 2016). A recent sedaDNA study (Wang et al. 2021) and subsequent computational modeling of the availability of suitable habitat (Fordham et al. 2024) have hypothesized a final disappearance of woolly rhinoceros as late as the early Holocene. We caution that other research has demonstrated that it is possible for DNA from extinct taxa to leach through permafrost (Seeber et al. 2024), which is important to take into consideration when assessing the isolated presence of extinct taxa in the sediment record. While it could be plausible that the woolly rhinoceros persisted beyond the currently recognized extinction date of ∼14 ka (Stuart and Lister 2012), especially in areas where habitat has been deemed to be favorable, further evidence where sedaDNA and modeling are combined with physical remains and other environmental proxies would be needed to corroborate a later extinction date. Thus, it remains most plausible that the extinction of woolly rhinoceros occurred rapidly, during the warming of the Bølling–Allerød interstadial.

## Conclusion

Here, we demonstrate the feasibility of recovering high-quality genomic data from poorly preserved material by generating a high-coverage woolly rhinoceros genome from around the time of its extinction. By analyzing Late Pleistocene genomes of woolly rhinoceros across the Late Pleistocene, we found no evidence of genomic erosion leading up to its extinction, with a relatively stable, albeit low, effective population size from ∼30 ka. The woolly rhinoceros' final decline toward extinction did not occur immediately at the onset of the Bølling–Allerød interstadial (14.7 to 12.8 ka) with a prolonged reduction in population size. We conclude that their decline toward extinction likely occurred rapidly after ∼14.4 ka, most likely driven by rapid changes in environmental conditions (Stuart and Lister 2012; Puzachenko et al. 2021).

## Supplementary Material

evaf239_Supplementary_Data

## Data Availability

All raw sequencing data is publicly available in the ENA project PRJEB77660.
