# Supplementary material for: Genome Shows no Recent Inbreeding in Near-Extinction Woolly Rhinoceros Sample Found in Ancient Wolf's Stomach
Source: Genome Biol Evol. 2026 Jan 14;18(1):evaf239. doi: 10.1093/gbe/evaf239 (PMC12799484; doi:10.1093/gbe/evaf239)
Supplement: evaf239_Supplementary_Data [file evaf239_supplementary_data.zip › GBE-240914-R1_stomach_rhino-suppl_info.pdf]

Supplementary information

Genome shows no recent inbreeding in near-extinction woolly rhinoceros sample found in ancient wolf’s stomach

S.M. Guðjónsdóttir, E. Lord *et al*.

List of supplementary figures

Figure S1. Picture of Tumat_14k sample.

Figure S2. Presence of Wolf DNA in the different Tumat_14k DNA extracts.

Figure S3. Principal component analysis using transversion-only dataset.

Figure S4. Distribution of Runs of Homozygosity (ROHs).

Figure S5. F_{ROH} estimates for all three samples (Tumat_14k, Pineyveem_18k and Rakvachan_49k) after removing transitions using BCFtools/RoH.

List of supplementary tables (attached separately as an Excel file)

Table S1. Sequencing information from all Tumat_14k DNA extracts.

Table S2. Sequencing summary statistics and metadata for the three genomes.

Table S3. Number of derived alleles out of 17888 derived sites identified in Lord et al. 2020

Table S4. Summary of Kraken Uniq results.

Table S5. ROH statistical tests.

Table S6. Genetic load analyses.

List of supplementary texts

Text S1. High-coverage shotgun sequencing

Text S2. Assessment of wolf DNA in Tumat_14k

Text S3. Metagenomic screening

Text S4. Additional analyses without removing transitions

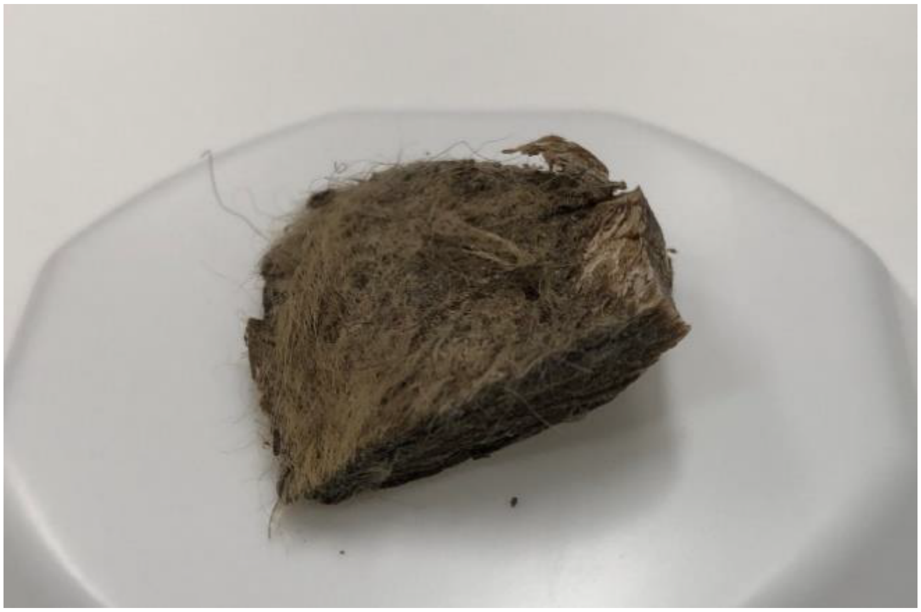

Figure S1. Tumat_14k specimen. Approximate size: 4 x 3 cm.

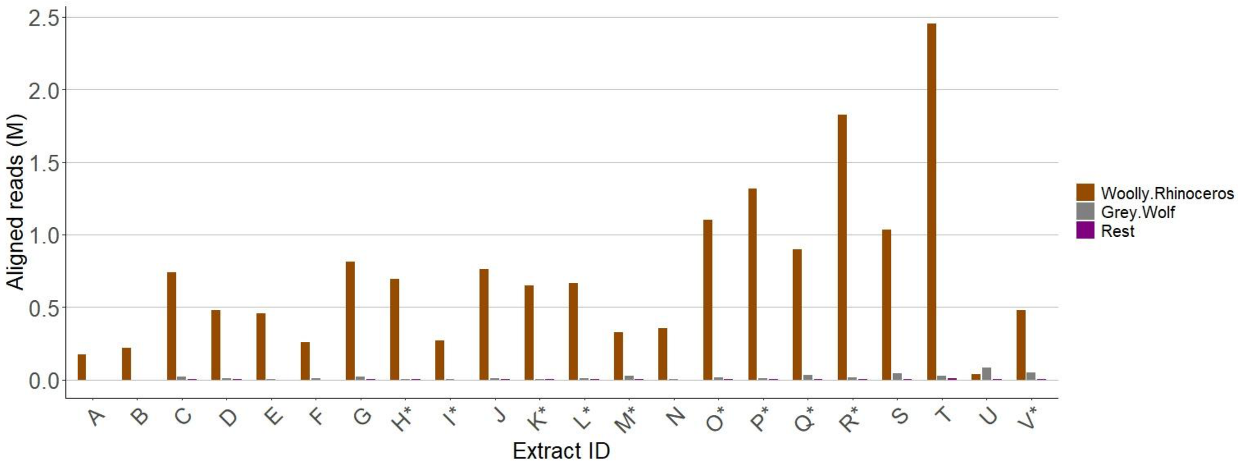

Figure S2. Presence of wolf DNA in the different Tumat_14k DNA extracts. The y axis shows the number of reads aligned to each reference mitogenome. For visualisation purposes, woolly rhinoceros and grey wolf are displayed separately while the rest (human, pig, cow, mouse and chicken) are merged into a single category. Extract U was excluded from all subsequent analyses. *Extracts used for the second round of sequencing.

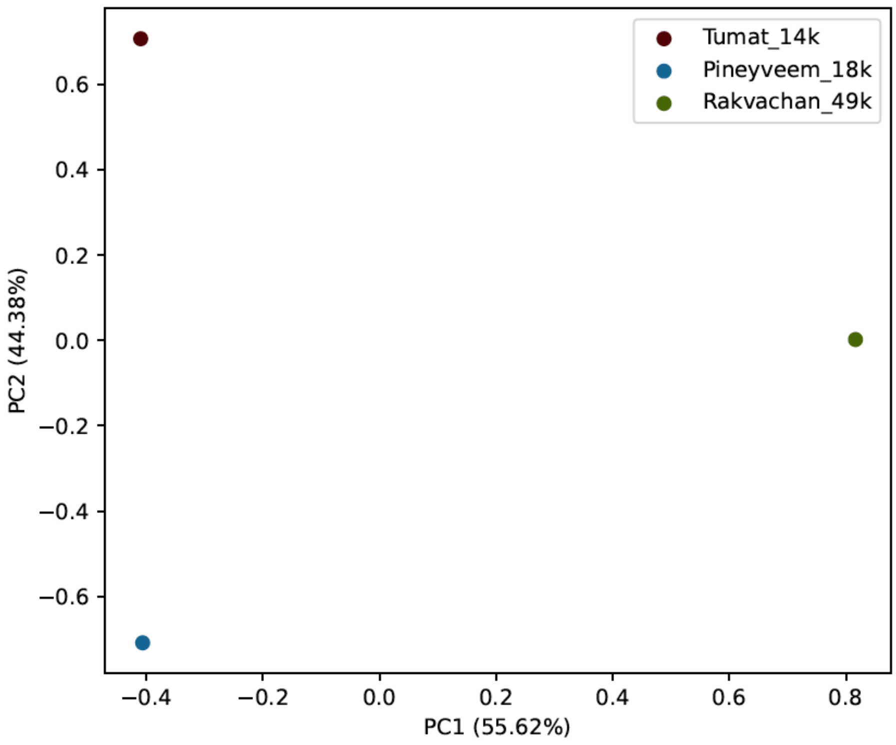

Figure S3. Principal component analysis using transversion-only dataset.

A)

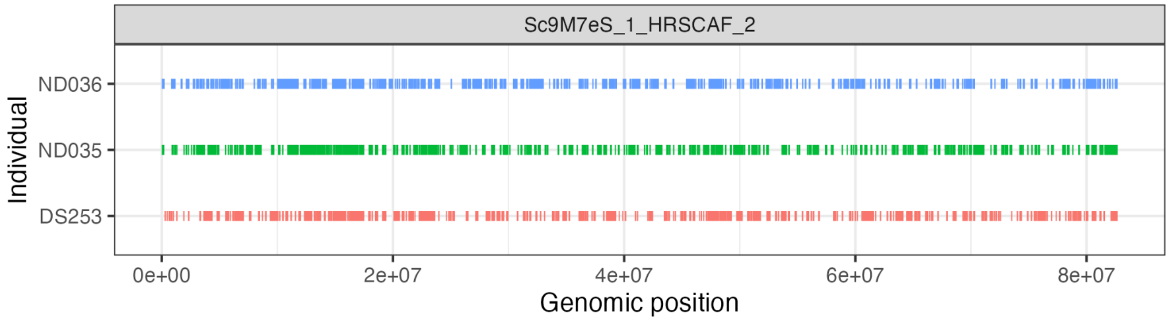

B)

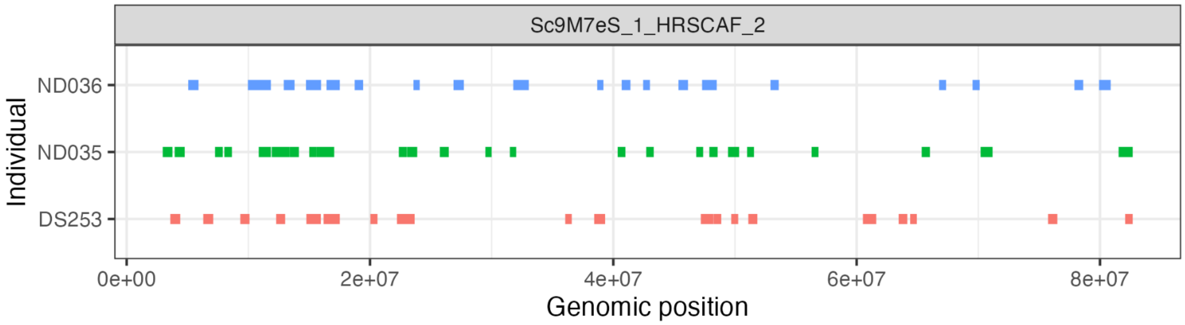

Figure S4. Distribution of Runs of Homozygosity (ROHs) **A)** above 100 kb and **B)** above 500 kb throughout scaffold Sc9M7eS_1_HRSCAF_2. ROHs were inferred using PLINK.

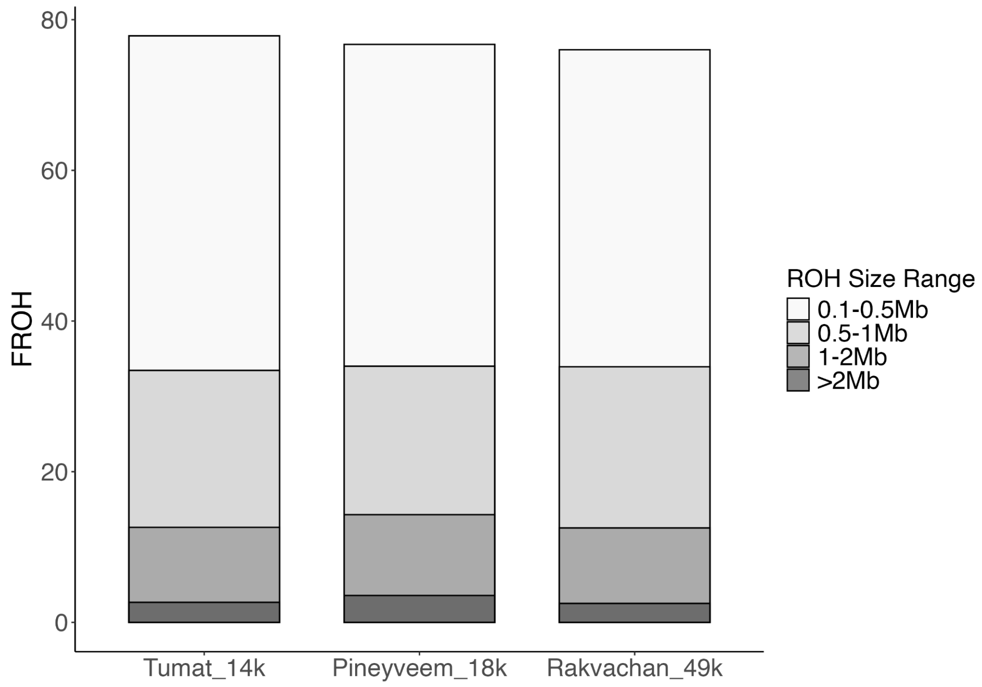

Figure S5. F_{ROH} estimates for all three samples (Tumat_14k, Pineyveem_18k and Rakvachan_49k) after removing transitions using BCFtools/RoH.

Text S1 - High-coverage shotgun sequencing

1.1 Generating a high coverage genome for Tumat_14k

The first round of sequencing of Tumat_14k (DS253) displayed considerable variation in DNA quality between the 20 different extractions (Table S1), with endogenous DNA content ranging from 1.9% to 8.3% and PCR duplicates from 15% to 42%. Endogenous content was estimated prior to duplicate removal as the percentage of total sequencing reads aligning to the reference genome. The low endogenous DNA content was expected due to the conditions in which the sample was preserved and as it had shown low levels on previous DNA extracts. We also assessed the genome recovery rate (GRR), which we defined as the portion of reads aligning to the reference genome after quality filtering steps, as well as removal of PCR duplicates and mitochondrial-linked scaffolds. Removing mitochondrial DNA to estimate the final GRR was important as mitogenomes occur in higher quantities than nuclear DNA within cells and could inflate the interpretation of endogenous content. The final portion of reads eventually used for downstream analyses (GRR) was below 5% for all the extracts after quality filtering steps.

Given the low GRR of the extracts, we made careful calculations for 10 extracts with the highest value to undergo additional library preparation and another round of sequencing to ensure enough high-quality endogenous DNA yield. We aimed for a 10X coverage genome to comparatively analyse it alongside two other high-coverage genomes. To achieve that, we first assigned a rough estimation of the depth of coverage we aimed to get from each of the 10 extracts from the second round of sequencing. Next, we calculated the number of reads required from each extract to reach that estimated coverage (Table 1) following the standard procedure used at the Centre for Palaeogenetics, demonstrated with equation 1 as

$$\frac{Reference\ genome\ length\ (bp)\ x\ desired\ coverage}{average\ read\ length\ (bp)\ x\ genome\ recovery\ rate} \quad (1).$$

In order to produce the number of reads needed from each extract, we prepared an additional library per extract and generated a total of 190 separate indexing PCRs (across 20 libraries) and estimated the concentration per sequencing library. When pooling together the libraries for the second round of sequencing, we estimated their final concentration in the pool by combining their estimated concentration with the relative contribution needed from each based on the number of reads desired. The amount of reads generated from the second round of sequencing for each extract was remarkably close to the estimated value.

Table 1: Overview of sequencing results for 20 of Tumat_14k extractions. Showing outcome from sequencing round 1 as total reads, complexity, number of reads from MQ30 uniq mapping to autosomes and genome recovery rate (final proportion of reads aligning to the reference genome after filtering steps; GRR). Desired number of reads from 10 extracts used in sequencing round 2 needed to reach desired coverage, compared with the total number of reads produced from the second round of sequencing.

Extract ID	Total reads from sequencing 1 (M)	Complexity (%)	Total MQ30 uniq reads mapped to autosomes (M)	Genome Recovery Rate (GRR %)	Reads for desired coverage (M)	Total reads from sequencing 2 (M)
**C**	96	65.47	0.9	0.85	-	-
**D**	71	67.92	0.6	0.91	-	-
**E**	65	85.08	1.1	1.67	-	-
**F**	64	73.28	0.5	0.81	-	-
**G**	77	64.36	1.3	1.69	-	-
**H**	87	80.71	2.7	3.05	1,380	1,286
**I**	57	81.11	1.4	2.39	432	380
**J**	104	73.55	2.0	1.93	-	-
**K**	92	65.01	2.3	2.47	419	396
**L**	63	72.22	2.2	3.47	1,453	940
**M**	63	69.81	1.5	2.36	432	542
**N**	55	81.30	1	1.81	-	-
**O**	99	79.21	3.9	3.92	1,519	1,484
**P**	110	57.61	2.6	2.36	439	339
**Q**	79	75.53	3.5	4.4	2,422	2,562
**R**	150	74.33	4.7	3.15	1,277	1,257
**S**	104	61.99	0.9	0.83	-	-
**T**	130	60.71	1.6	1.26	-	-
**U**	72	67.75	0.7	0.96	-	-
**V**	113	71.86	3.5	3.06	1,452	1,874

1.2 Samples & sequencing results

By combining all sequencing for Tumat_14k, the sample reached an average of 10.1X, comparable with the other two samples: 11X for Pineyveem_18k and 11.1X for Rakvachan_49k (Table S2). The coverage for the latter two is lower than reported in their original publications (Lord et al. 2020; Liu et al. 2021), likely because a more stringent quality filtering was applied in this analysis, including a second round of duplicate removal. The final endogenous DNA content for Tumat_14k was 5%, low compared to Pineyveem_18k and Rakvachan_49k with 56% and 35%, respectively. The overall number of duplicates was nearly twice as high for the stomach rhino sample. Aiming for higher sequencing depth could come at the cost of reduced complexity as unique fragments can get exhausted from the sample (Dehasque et al. 2022).

Text S2 - Assessment of wolf DNA in Tumat_14k

Since Tumat_14k was found in the stomach of a grey wolf, it was essential to assess the extent of wolf DNA in the sequencing data. Ancient samples often contain other DNA sources as well, so we used a competitive mapping (alignment) approach (Feuerborn et al. 2020) where the original merged reads were aligned to multiple mitogenomes. We created a concatenated fasta file using the reference mitogenomes of the woolly rhinoceros (*Coelodonta antiquitatis*, NC_012681.1), grey wolf (*Canis lupus*, NC_008092.1), human (*Homo sapiens*, NC_012920.1), pig (*Sus scorfa*, NC_000845.1), cow (*Bos taurus*, NC_006853.1), mouse (*Mus musculus*, NC_005089.1) and chicken (*Gallus gallus*, NC_001323.1). Each extract was aligned using the same approach described in the main text's methods section "Alignment of sequencing data". The results were analysed using SAMtools v1.17 idxstats which gave the number of reads that aligned to each mitogenome. For the competitive mapping, we used the reads from sequencing round 1 for extracts C-V as well as the fastq file from the published extract A (Lord et al. 2020) and a subsampled fastq file for extract B.

Most of the sample's extracts had minimal wolf DNA with under 5% of the aligned reads corresponding to the grey wolf mitogenome and under 0.4% to the rest of possible DNA sources, including humans (Fig S2). However, extract U displayed a high amount of wolf DNA, with 66% of reads aligning to the grey wolf mitogenome and only 31% to the woolly rhinoceros mitogenome. This extract was subsequently excluded from all downstream analyses to reduce the risk of wolf DNA bias in the dataset. As the analyses were conducted on a high-coverage genome, the chance of calling false SNPs (single nucleotide polymorphisms) due to wolf DNA bias in the dataset is minimal (Llamas et al. 2017; Renaud et al. 2019).

Nonetheless, since the mitogenome-based estimates potentially only offer a lower bound for the estimation of the amount of contamination present on the sequencing data and to corroborate that this contamination does not affect our inferences, we also performed a competitive mapping using the entire reference genomes for the target species (Sumatran rhinoceros) and the main source of contamination (grey wolf). We followed exactly the same procedures described in the main text (see methods sections "Data processing" and "Variant calling"), with the only difference that we excluded all reads mapping to grey wolf prior to variant calling.

Across the 20 extracts, an average of ~1% of the sequenced reads mapped to the grey wolf reference genome. After removing PCR duplicates and filtering for mapping quality 25, only ~0.03% of the sequenced reads aligned to grey wolf. Table 1 (below) shows the contamination estimations for each extract as estimated from the same screening round described in Text S1. We additionally included a library from ND036 (Rakvachan_49k) to compare the base levels of DNA mapping to grey wolf in a sample that theoretically should not contain any contamination of this kind, providing a baseline.

Table 1. Contamination estimates obtained from the competitive mapping approach using the concatenated sumatran rhinoceros and grey wolf reference genomes

Extract ID	% of total reads mapped to grey wolf	% of total reads mapped to grey wolf after MQ25
A	6.332	0.009
B	0.217	0.01
C	0.564	0.031
D	0.386	0.022
E	0.26	0.017
F	0.501	0.029
G	1.266	0.043
H	0.606	0.018
I	0.661	0.021
J	0.774	0.019
K	1.593	0.019
L	1.529	0.029
M	1.13	0.06
N	0.507	0.02
O	0.864	0.029
P	2.155	0.025
Q	1.388	0.059
R	0.714	0.021
S	0.733	0.053
T	0.636	0.03
U	0.743	0.131
V	1.018	0.058
ND036_08_L1	0.237	0.021

After variant calling, we obtained a genome coverage of 9.9x for Tumat_14k, almost identical to the one originally obtained. To corroborate that our estimations hold regardless of the alignment method used (from now on regarded as “non-competitive” and “competitive”) we subsampled the non-competitive based Tumat_14k genome from 10.1x to 9.9x and estimated genome-wide heterozygosity using direct counts from BCFtools (following the same procedures described in the main methods section. We got an estimated ~1.2 SNPs per 1,000bp for both approaches (including all types of variants). This demonstrates that wolf contamination does not have an effect on variant calling and downstream analyses.

Text S3 - Metagenomic screening

To assess the presence of ancient host-associated microbes and pathogens, we performed a metagenomic screening with the first module of the aMeta pipeline (commit 16554c6)(Pochon et al. 2023). In summary, it performs a quality control check with FastQC (https://www.bioinformatics.babraham.ac.uk/projects/fastqc/), before and after adapter removal with Cutadapt(Martin 2011). Then it runs KrakenUniq(Breitwieser et al. 2018) to classify the reads using the aMeta Microbial NCBI NT database, available on https://doi.org/10.17044/scilifelab.20518251. This database comprises all microbial genomic information from NCBI NT (viruses, bacteria, archaea but also eukaryotic microbes like fungi, protozoa and parasitic worms) and a few other eukaryotic complete genomes. Subsequently, KrakenUniq outputs are filtered to keep species that have at least 200 reads and 1,000 *k*-mers.

In parallel, aMeta performs an alignment to a microbial-human genome database https://doi.org/10.17044/scilifelab.21185887 using Bowtie2(Langmead & Salzberg 2012) and it creates deamination plots for the authentication of the microbial ancient status with mapDamage2(Jónsson et al. 2013). Since two out of three samples were USER-treated, we also used PMDtools(Skoglund et al. 2014) to estimate post-mortem damage based on CpG sites.

Interestingly, we could not identify any ancient microbe in the untreated rhino sample. Furthermore, investigation of a deamination profile based on CpG sites for USER-treated samples did not reveal ancient microbial organisms either, maybe due to lack of coverage, but the presence of mostly modern contaminants cannot be excluded (Table S4).

Several organisms were found in two out of three samples but are interpreted as environmental contamination like *Cupriavidus metallidurans*, *Cutibacterium acnes*, *Enterococcus faecalis*, *Herbaspirillum seropedicae*, *Paeniclostridium sordellii*, *Rhodopseudomonas palustris*, *Staphylococcus epidermidis*, *Streptococcus canis* and *Variovorax paradoxus*. Although generally considered an opportunistic pathogen in dogs, *S. canis* was identified in both DS253 and ND035 making it more likely to be a sign of contamination. Additionally, *Collimonas spp.*, *Dictyostelia spp.* (amoeba), *Jonesia denitrificans*, *Sphingomonas melonis* and *Pseudomonas yamanorum* are associated with soil. Furthermore, *Clostridia spp.*, *Listeria monocytogenes* and *Paraclostridium bifermentans* are associated with the intestinal tract of animals but are also commonly found in soils. Moreover, *Streptococcus pyogenes* might be due to human contamination. Finally, the *Carnobacteria* and *Lactobacilli* species found are generally associated with meat kept in cold environments.

Text S4 - Additional analyses without removing transitions

Demographic analyses

PSMC was performed with and without transitions (Figure 1). For both analyses we also removed the last 10,000 years (6 steps).

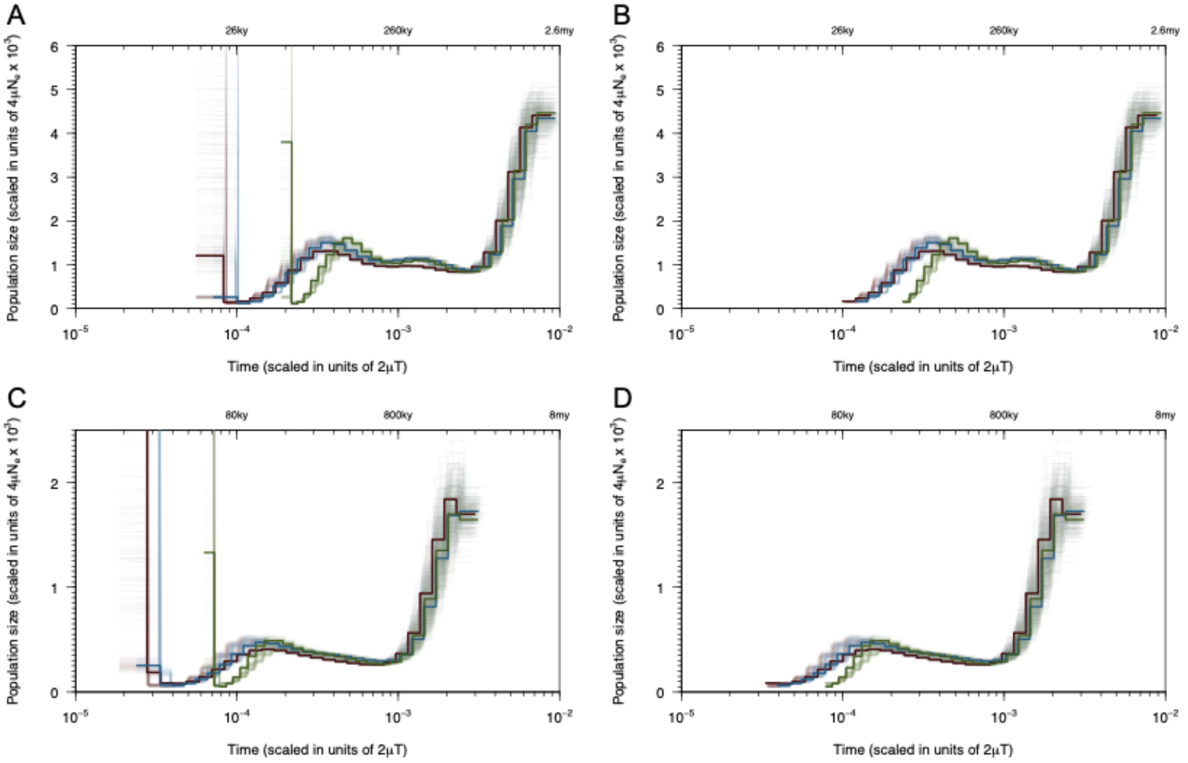

Figure 1: All plots show DS253 in red, ND035 in blue, and ND036 in green, with 300 bootstrap replicates in pale shades. **A** shows the PSMC including transitions, scaled using a mutation rate of 2.34x10-8 substitutions per site per generation and a generation time of 12 years. **B** shows the PSMC including transitions, scaled using a mutation rate of 2.34x10-8 substitutions per site per generation and a generation time of 12 years with the most recent 10000 years removed. **C** shows the PSMC excluding transitions, scaled using a mutation rate of 0.78x10-8 substitutions per site per generation and a generation time of 12 years. **D** shows the PSMC including transitions, scaled using a mutation rate of 0.78x10-8 substitutions per site per generation and a generation time of 12 years with the most recent 10,000 years removed.

Heterozygosity and inbreeding

The downstream analyses were also performed using with and without transitions. For the two younger samples the estimated population mutation rate (θ) was 1.77 SNPs per 1,000bp (95% CI: 1.77-1.77) for Tumat_14k and 1.64 (95% CI: 1.64-1.65) for Pineyveem_18k. Additionally we estimated genome-wide heterozygosity using allele counts from variant calling which revealed ~1.2 SNPs per 1,000bp for both samples. These two estimates differ due to different approaches in estimating heterozygosity, with θ accounting for possible sequencing error rate and recombination within the genome(Haubold et al. 2010). The estimates for the younger two samples decreased

after removing transitions from the genome, as they rely solely on heterozygous sites that occur from transversions (around ⅓ of total SNPs).

By including transitions in inbreeding estimations, Tumat_14k and Pineyveem_18k had 41% and 42% of their genome within homozygous segments, respectively. The longest ROH segment was 5.2 Mb for Tumat_14k and 4.7 for Pineyveem_18k. For both samples, 98% of all ROH windows were under 1 Mb long.

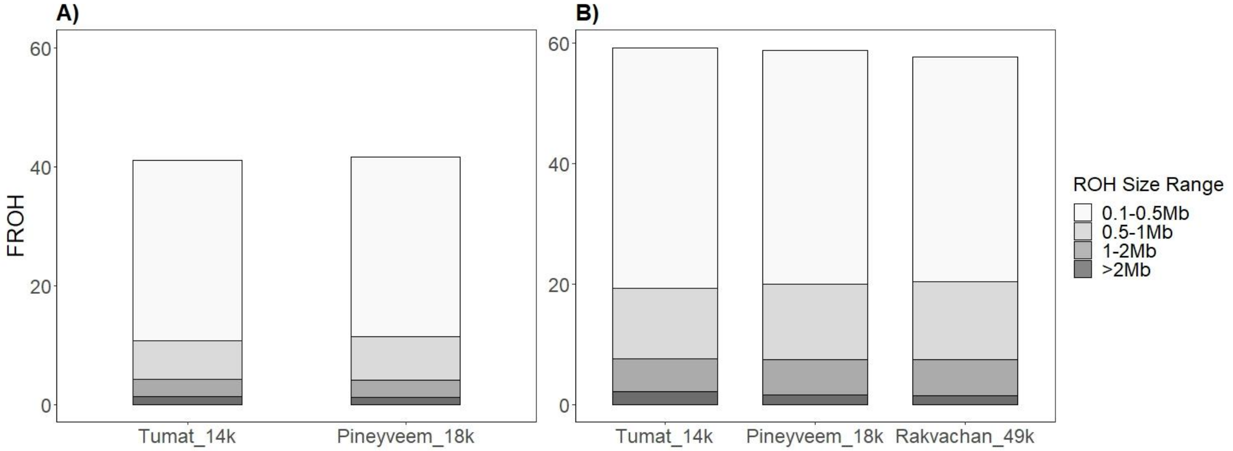

Figure 2: A) F_{ROH} results for the two younger samples with transitions/transversions B) F_{ROH} for all three samples (Tumat_14k, Pineyveem_18k and Rakvachan_49k) after removing transitions. White and grey coloured markings show the F_{ROH} value for short segments while black indicates long ROH window sizes >2Mb.

Statistical tests

We tested for differences in the average size of ROHs among the samples for four different size thresholds >0.1Mb, >0.5Mb, >1Mb and >2Mb. As the ROH window sizes followed a non-normal frequency distribution, non-parametric statistical tests were used. We used the Wilcoxon-rank-sum U test for the two younger samples including transitions (Table 2) and the Kruskal-Wallis test for all three samples with transversions only (Table S3). The null hypotheses H_0 in both cases were that the frequency distributions of ROH sizes were equal between samples and were conducted using R v.4.2.3(R Core Team 2023). As the tests were being applied simultaneously on a single dataset, the Bonferroni Correction was applied to avoid generating false-positives(Dunn 1961). With a 99% confidence interval, the critical p-value was set as 0.01, and as we were analysing four different size thresholds for two types of statistical tests simultaneously, the Bonferroni Correction set the critical p-value to 0.01/8 = 0.001 (Table S3).

Table 2: Summary results comparing different sizes of Runs of Homozygosity (ROH).

**ROH length threshold**	**> 0.1Mb**	**>0.5Mb**	**>1Mb**	**>2Mb**
**DS253-ND035**<br>**Wilcoxon U test (p-value)**	0.031	0.635	0.405	0.173
**DS253**				
Nr. of ROHs	3,850	288	60	8
Mean length (Mb)	0.25	0.86	1.62	3.75
Median length (Mb)	0.17	0.69	1.29	4.01
**ND035**				
Nr. of ROHs	3,820	314	65	10
Mean length (Mb)	0.25	0.84	1.48	2.8
Median length (Mb)	0.18	0.7	1.23	2.64

References

Breitwieser FP, Baker DN, Salzberg SL. 2018. KrakenUniq: confident and fast metagenomics classification using unique k-mer counts. Genome Biol. 19:198.

Dehasque M et al. 2022. Development and Optimization of a Silica Column-Based Extraction Protocol for Ancient DNA. Genes . 13. doi: 10.3390/genes13040687.

Dunn OJ. 1961. MULTIPLE COMPARISONS AMONG MEANS. J. Am. Stat. Assoc. 56:52–&.

Feuerborn TR et al. 2020. Competitive mapping allows for the identification and exclusion of human DNA contamination in ancient faunal genomic datasets. BMC Genomics. 21. doi: 10.1186/s12864-020-07229-y.

Haubold B, Pfaffelhuber P, Lynch M. 2010. mlRho - a program for estimating the population mutation and recombination rates from shotgun-sequenced diploid genomes. Mol. Ecol. 19:277–284.

Jónsson H, Ginolhac A, Schubert M, Johnson PLF, Orlando L. 2013. mapDamage2.0: fast approximate Bayesian estimates of ancient DNA damage parameters. Bioinformatics. 29:1682–1684.

Langmead B, Salzberg SL. 2012. Fast gapped-read alignment with Bowtie 2. Nat. Methods. 9:357–359.

Liu SL et al. 2021. Ancient and modem genomes unravel the evolutionary history of the rhinoceros family. Cell. 184:4874–+.

Llamas B et al. 2017. From the field to the laboratory: Controlling DNA contamination in human ancient DNA research in the high-throughput sequencing era. STAR: Science & Technology of Archaeological Research. 3:1–14.

Lord E et al. 2020. Pre-extinction Demographic Stability and Genomic Signatures of Adaptation in the Woolly Rhinoceros. Curr. Biol. 30:3871–+.

Martin M. 2011. Cutadapt removes adapter sequences from high-throughput sequencing reads. EMBnet.journal. 17:10–12.

Pochon Z et al. 2023. aMeta: an accurate and memory-efficient ancient metagenomic profiling workflow. Genome Biol. 24:242.

R Core Team. 2023. R: A language and environment for statistical computing. https://www.R-project.org/.

Renaud G, Hanghoj K, Korneliussen TS, Willerslev E, Orlando L. 2019. Joint Estimates of Heterozygosity and Runs of Homozygosity for Modern and Ancient Samples. Genetics. 212:587–614.

Skoglund P et al. 2014. Separating endogenous ancient DNA from modern day contamination in a Siberian Neandertal. Proc. Natl. Acad. Sci. U. S. A. 111:2229–2234.
